# Behavior of volcanic ash–soil mixtures under one-dimensional compression testing

**DOI:** 10.1038/s41598-022-18767-8

**Published:** 2022-08-25

**Authors:** Mohammad Amin Sayyah, Saeed Abrishami, Pooya Dastpak, Daniel Dias

**Affiliations:** 1 Department of Civil Engineering, Faculty of Engineering, Ferdowsi University of Mashhad, Mashhad, Iran; 2 Department of Civil, Environmental, and Ocean Engineering, Stevens Institute of Technology, Hoboken, NJ 07030 USA; 3 Univ. Grenoble Alpes, CNRS, Grenoble INP, 3SR Lab, 38000 Grenoble, France

**Keywords:** Environmental sciences, Engineering, Civil engineering, Structural materials

## Abstract

Volcanic ashes (VA) are one of the by-products of explosive volcanic eruptions. They can be used as a soil stabilizer due to their cementitious properties as an eco-friendly soil stabilization approach. In this study, the impact of VA as an additive material (up to 20%) was investigated on the behavior of a clayey soil under one-dimensional compression tests and uniaxial compression tests. To this aim, the VA percentage effect, curing conditions, i.e. the optimum moisture content (OMC) and saturated sample, and curing time, on the oedometer modulus, and the uniaxial compression strength (UCS) are investigated. Results show that the addition of VA increases the UCS continuously in saturated conditions. However, this improvement is considerable for 5% additional VA at the OMC state and it induces 325% improvement in UCS. The maximum improvement of UCS occurs at 20% addition of VA in saturated condition. It was also revealed that VA-soil mixtures are more sustainable at low stress levels and the oedometer modulus increases with the VA addition. A long-term curing time leads to an increase of the fabricated bonds due to the pozzolanic reaction. Additional VA has no significant effect on the consolidation parameters specifically for short-term curing time.

## Introduction

During recent decades, soil improvement and stabilization of road construction projects have considered eco-friendly approaches regarding the restrictions and available sources. The adopted solutions consider the use of geosynthetics^1–3^, the addition of polymer/biopolymer to the soils^4–6^, or, the addition of different sorts of waste materials such as tires or, crushed glass^7–11^. Based on previous studies, soil stabilization is a significant and cost-effective method for the road sustainability^12^. Chemical improvement of soils is one of the most common techniques which can be in accordance with the environment and project expenses. Nowadays, chemical improvements are performed by using cementitious materials such as cement, lime and, pozzolans^12–16^.

Even though cement and lime have high performance and efficiency in soil stabilization, several environmental problems such as degradation and pollution can be caused during the manufacturing process or usage^12,17^. Throughout recent years, regarding the problems associated with cement and lime mixture in soils, using environmentally friendly waste materials became widespread among engineers. Noting the previous efforts, using pozzolans as a soil improvement material becomes a popular topic among investigators since it has relatively no negative environmental issue^12,17^. Pozzolans are siliceous based materials which are diversified according to the way they are produced (fly ashes, volcanic ashes, husk ashes, or others). Using pozzolans can be productive for the air pollution reduction as well as saving energy and greenhouse emissions^17,18^.

The potential of using waste materials produced by agricultural fields and industrial firms namely, fly ashes, slags, tires, glasses, and grain shells were recently investigated^8,19–26^. Fly ashes and Volcanic Ashes (VA) which are respectively wasted by coal-fired furnaces and volcanic eruptions can be chosen as an appropriate replacement of cement/lime and they can also be used to stabilize soil. Numbers of researchers have studied the behavior of VA under different conditions. In order to evaluate the soil improvement using additional cementitious materials, the bonding between the particles, and strength properties should be estimated^27^. To this aim, standard conventional tests can be performed such as direct shear tests, triaxial tests, uniaxial, confined compression tests, and California Bearing Ratios (CBR). Adding pozzolans to the soil mass can improve the soil properties such as strength, friction angle, cohesion and, CBR^21–33^.

Liu et al.^28^ added fly ash-based geopolymers to stabilize loess soils. The ratio between the fly ash and loess soil used in their study were equal to 10%, 20%, and 30%. With increasing the fly ash proportion, both young modulus and compression strength increase. Xiao et al.^29^ investigated the impact of additional fly ash on the improvement of marine clays by uniaxial compression tests. The improvement significantly depends on the curing period, fly ash content, and water content. They stated that for the specimen with the higher fly ash content (34.5%) and 90–150 curing days, the compression strength highly increases (up to 85%). Furthermore, compared to ordinary Portland cement, the effectiveness of a long curing period is greater when fly ashes are used as an additive material^29^. Mir and Sridharan^30^ studied the effect of additional fly ashes on the compressibility index of clayey soil mixtures using one-dimensional compression tests at three different curing times: 1, 7, and 28 days. For the long-term condition, the optimum percentage of additional fly ash was approximatively equal to 20% for the compressibility properties improvement. However, the optimum fly ash content was equal to 60% for the short-term period. According to the results obtained by Ma et al.^31^, using fly ash as a stabilizer increases the secant modulus as well as the compression strength by increasing the curing time.

Solanki et al.^32^, Pinilla et al.^33^, and Edil et al.^34^ used fly ash as an additive and conducted several CBR tests to determine the resilient modulus. The addition of fly ash leads to a significant increase of the resilient modulus. Moreover, the resilient modulus behavior has a nonlinear relationship with curing time as well as with moisture content. Pandian and Krishna^35^ addressed the impact of two different kinds of fly ashes on CBR tests (class C and class F). Fly ash allowed to improve the mechanical resistance. The difference between these two types of fly ashes is related to the amount of calcium. This substance plays an important role in pozzolanic reactions^36^. Turner^37^ used fly ash as a stabilizer material for aggregate-surfaced roads. Additional fly ash has a significant effect on the strength parameters of the subgrade. The effect considerably depends on the water/stabilizer ratio. It was also observed that fly ash addition led to the design thickness decrease about five times which then increase the cost effectiveness.

Various investigations conducted on the application of VA alone or incorporating them with lime or cement in different pavement layers, subgrades, capping layers, or subbases. Bahadori et al.^27^ used three different VAs, extracted from different regions, to stabilize marly soils. The soil plasticity index decreased by adding VA considerably. The elasticity modulus determined by uniaxial compression tests increased. Hossain et al.^12^ and Hossain and Mol^38^ stabilized clayey soils using VA. The durability of the stabilized soil mass was evaluated by investigating the soaking impact on strength, water absorbability, and drying shrinkage. Adding a high percentage of VA (up to 20%) has a greater impact than using a combination of cement and VA. Hastuty and Ramadhany^39^ performed CBR and uniaxial tests to assess the VA addition on the clay performance. VA-soil mixtures have a higher strength and CBR values. Iskandar et al.^40^ carried out several CBR and uniaxial tests. They observed that the addition of VA and gypsum improves both the CBR and uniaxial strength of more or less 100%.

The downtown of Mashhad, (capital of Khorasan Razavi, a province of Iran) contains decayed clayey soils that are composed of fine particles. These fine-grained soils have poor properties such as weak strength, lack of appropriate strength, and low stiffness. Therefore, a soil stabilization is required to ensure the sustainability and displacements led by external loads due to constructions.

As indicated in the literature, the majority of investigations are related to the fly ash soil stabilization and the effect of additional VA is not well investigated. Only a few efforts have addressed the effectiveness of VA on the mechanical properties of soils^12,33–35,39^. Compressibility, and consolidation behavior of the stabilized clay as subgrade materials are critical issues for infrastructures construction. Consolidation behavior of VA-soil mixture was studied so far. Thus, the primary goal of this study is to understand the consolidation behavior of the VA-soil mixture in two possible curing conditions namely the Optimum Moisture Content (OMC) and saturated conditions for 7, 14, 28, and 90 days. In addition, the oedometric properties of the VA-soil mixture are determined for the first time in this study. The oedometric properties are often used for settlements estimation and numerical modeling. Also, the consolidation parameters namely the compression index (C_c_), swelling coefficient (C_s_), and recompression index (C_r_) for the saturated conditions are measured for the VA-soil mixture at different curing times. Last but not least, the oedometer elasticity modulus of the VA-soil mixture is determined at different stress levels, from 25 to 400 kPa. This parameter is the most important one for the settlements estimation. To this aim, several standard tests namely, proctor tests, one-dimensional compression, and uniaxial compression tests were conducted. Four proportions of VA, 5%, 10%, 15%, and 20%, are mixed with clayey soils. The samples were prepared and cured at the OMC and saturated conditions representing the in-site project limitations namely weather condition. Uniaxial Compression Strength (UCS) at both OMC and saturated conditions are determined. Also, Scanning Electron Microscopy (SEM) and Energy Dispersive Spectroscopy (EDS) were used to show the bond formations before and after treatment.

## Materials

### Clayey soil

**Figure 1:**
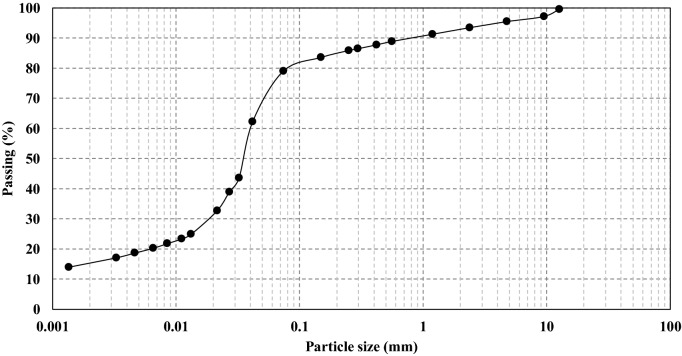
Soil grain size distribution.

The soil used in this research was extracted from the center of Mashhad—Iran. The soil physical and mechanical properties were determined according to the American Society for Testing and Materials (ASTM)^41–43^. These properties are presented in Table 1. The soil is classified as CL-ML as per Unified Soil Classification System (USCS)^44^ and as A-6 according to the AASHTO classification standard (ASTM D3282)^45^. Figure 1 shows the soil grain size distribution curve. Table 2 describes its chemical properties and elements. The maximum amount of chemical compound is CaCO_3_ with 14.55%. CaO is produced by the water presence within the soil having CaCO_3_ compounds. Consequently, additional pozzolans containing either SiO_2_, Al_2_O_3_, or Fe_2_O_3_ compounds will react with the produced CaO, and will create Calcium Silicate Hydrate (CSH) and Calcium Aluminate Hydrate (CAH) which causes the soil improvement^27^.

**Table 1:** Soil properties.

Soil properties	Value
Specific gravity	2.7
**Grain size analysis (%)**	
Gravel	4.5
Sand	16.4
Silt	63.6
Clay (particle size < 0.002 mm)	15.5
**Consistency limit**	
Liquid limit	26.0
Plastic limit	21.1
Plastic index	4.9
USCS classification	CL-ML
AASHTO classification	A-6

**Table 2:** Chemical properties of the clayey soil.

pH	7.95
CL (%)	0.02
SO_4_ (%)	0.1
CaCO_3_ (%)	14.55
MgCO_3_ (%)	2.01

### Volcanic ash (VA)

**Figure 2:**
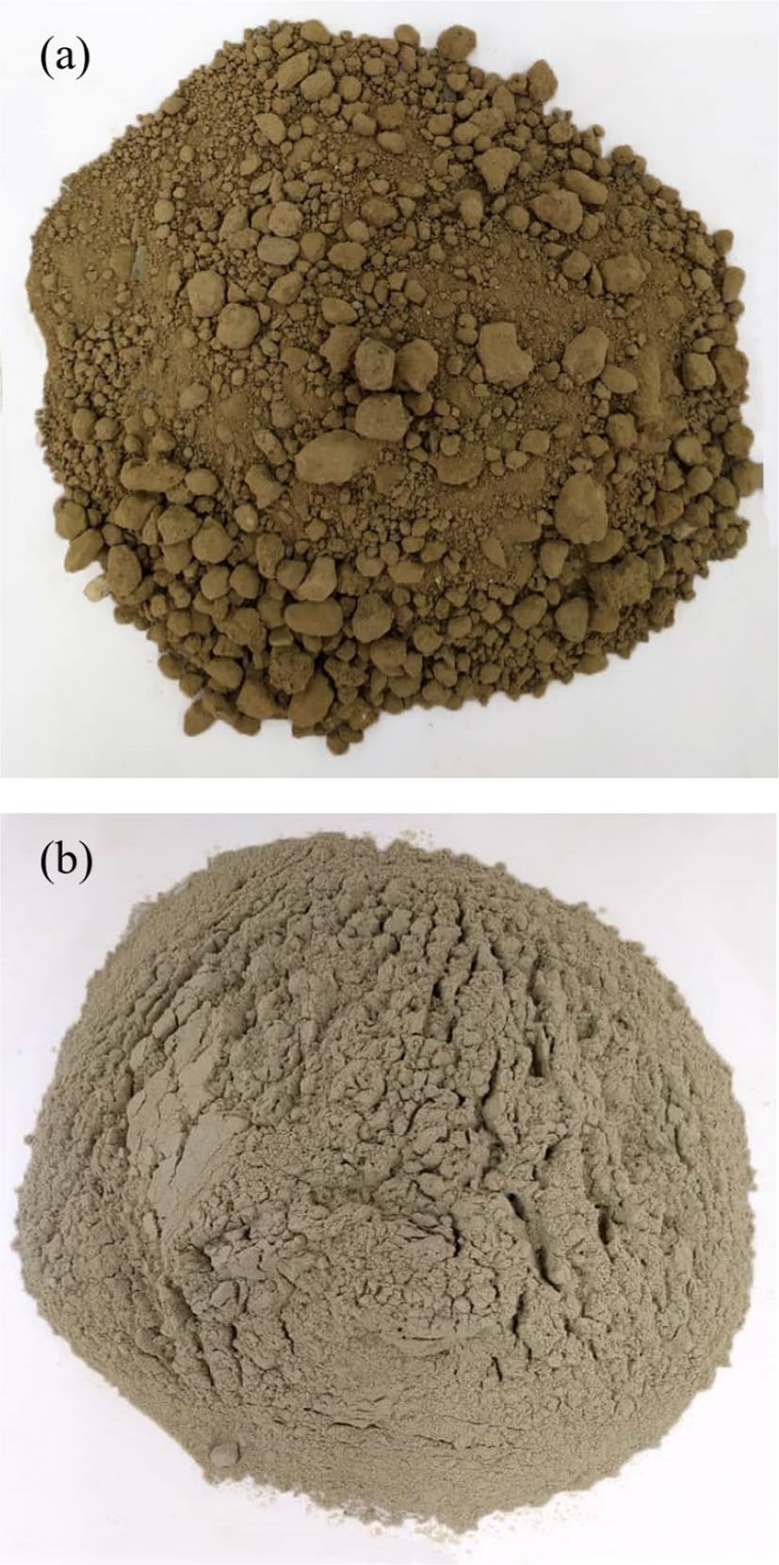
Materials used in this research: (**a**) clayey soil, (**b**) volcanic ash (VA).

**Figure 3:**
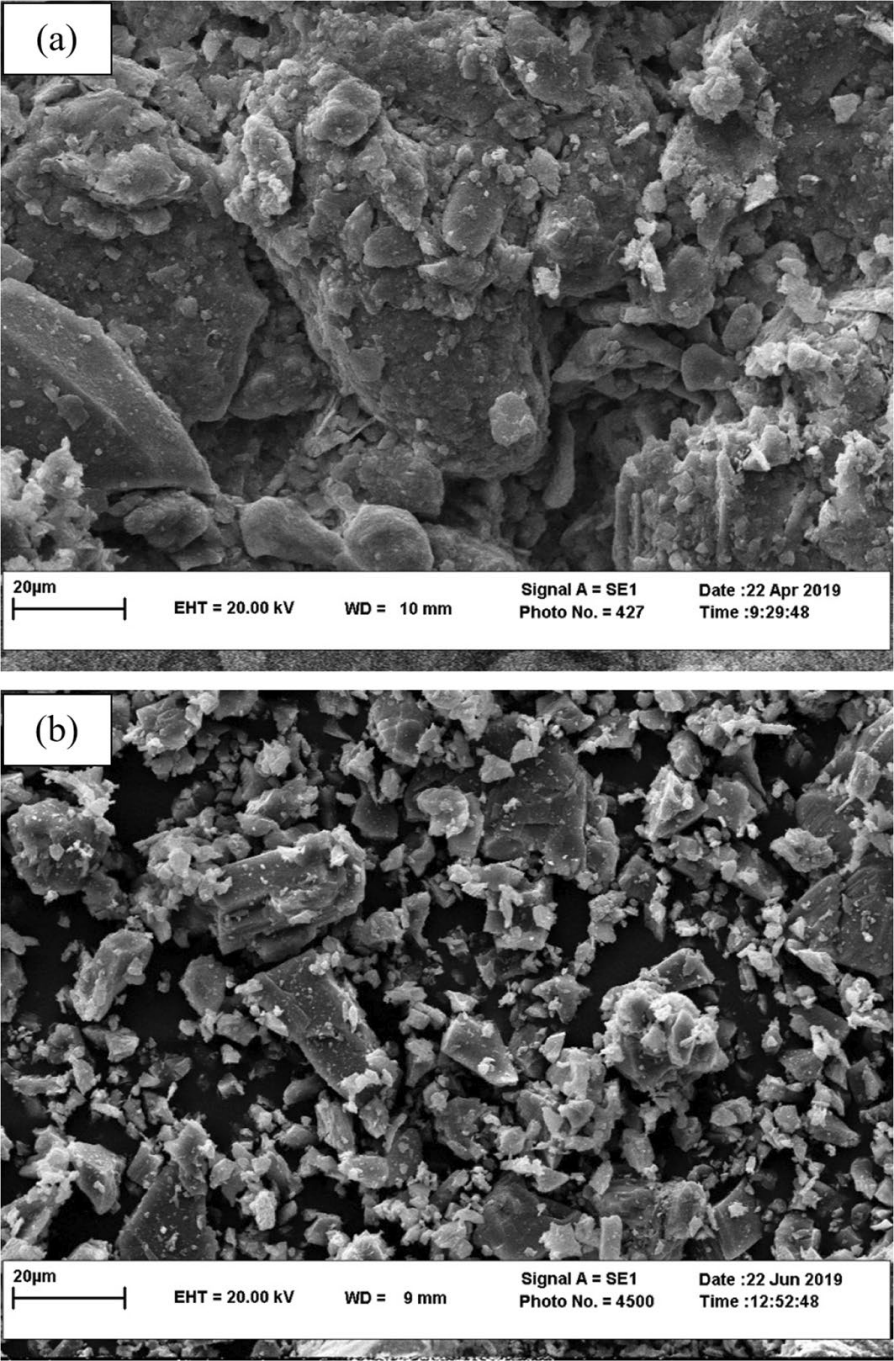
SEM micrographs of used materials; (**a**) clayey soil, (**b**) volcanic ash (VA).

The pozzolan used in this research was extracted from a natural pozzolan mine located at 140 km northwest of Mashhad in Iran. The coarser particles were passed throughout a crusher machine and a #200 sieve to assure that the VAs particle sizes are fine enough. The specific gravity (G_s_) of the VA is 2.05 based on the ASTM D854-14^43^. It is classified as a N pozzolanic material according to ASTM C618^46^ standard. Table 3 describes the chemical compounds and elements of the VA estimated according to the X-Ray Diffraction (XRD) technique. Figure 2 illustrates the soil and VA used in this study. Figure 3 shows the Scanning Electron Microscope (SEM) photos of the soil and VA. In terms of grain size and shape, the soil sample (Fig. 3a) has larger and smoother particles compared to VA (Fig. 3b). In fact, due to the shattering process, VA has angular particle shapes.

**Table 3:** Chemical compounds and elements of the volcanic ash (VA).

Chemical composition	Natural pozzolan (%)
SiO_2_	69.54
Al_2_O_3_	15.68
CaO	3.77
Fe_2_O_3_	2.30
K_2_O	2.22
MgO	0.83
MnO	0.09
Na_2_O	4.60
P_2_O_5_	0.11
TiO_2_	0.29
LOI	0.48

## Experimental program

A series of standard tests including compaction, uniaxial compression, and one-dimensional compression tests were conducted to investigate the VA addition effect on the soil improvement performances.

These tests were carried out considering various curing times and percentages of VA. It is worth noting that compaction and uniaxial tests were performed for validation and control of the improvement procedure. In order to study the oedometric parameters and consolidation parameters, one-dimensional compression tests were conducted. A period 28 days was considered for the curing time in the uniaxial compression tests. In terms of one-dimensional compression tests, Table 4 summarizes the varied parameters and the testing program. The general designation of each test is named VA_n_D_t_-X in which, VA_n_ indicates the n VA-soil mixture percentage, D_t_ the t curing time days, and X the curing conditions. VA_0_, VA_5_, VA_10_, VA_15_, and VA_20_ respectively stand for 0, 5, 10, 15, 20% of VA-soil mixtures. These mixtures are based on previous investigations^31^. The curing procedure is done at the Optimum Moisture Content (-OMC) or in Saturated (-S) conditions in 7, 14, 28, and 90 days. For instance, VA_5_D_7_-S represents 5% of VA in 7 curing days considering saturated conditions. It should be noted that all the tests were performed at a relative standard proctor compaction (RC) of 90%.

**Table 4:** Testing program.

Soil (%)	Volcanic Ash, VA (%)	Curing condition	Curing period (day)	Designation
100	0	OMC	0	VA_0_D_0_-OMC
		Saturated	0	VA_0_D_0_-S
95	5	OMC	7	VA_5_D_7_-OMC
			14	VA_5_D_14_-OMC
			28	VA_5_D_28_-OMC
			90	VA_5_D_90_-OMC
		Saturated	7	VA_5_D_7_-S
			14	VA_5_D_14_-S
			28	VA_5_D_28_-S
			90	VA_5_D_90_-S
90	10	OMC	7	VA_10_D_7_-OMC
			14	VA_10_D_14_-OMC
			28	VA_10_D_28_-OMC
			90	VA_10_D_90_-OMC
		Saturated	7	VA_10_D_7_-S
			14	VA_10_D_14_-S
			28	VA_10_D_28_-S
			90	VA_10_D_90_-S
85	15	OMC	7	VA_15_D_7_-OMC
			14	VA_15_D_14_-OMC
			28	VA_15_D_28_-OMC
			90	VA_15_D_90_-OMC
		Saturated	7	VA_15_D_7_-S
			14	VA_15_D_14_-S
			28	VA_15_D_28_-S
			90	VA_15_D_90_-S
80	20	OMC	7	VA_20_D_7_-OMC
			14	VA_20_D_14_-OMC
			28	VA_20_D_28_-OMC
			90	VA_20_D_90_-OMC
		Saturated	7	VA_20_D_7_-S
			14	VA_20_D_14_-S
			28	VA_20_D_28_-S
			90	VA_20_D_90_-S

## Sample preparation and testing procedure

The dried soil was mixed with the desired percentage of VA in dry conditions. A homogenous mix of the VA-soil was prepared by adding water and then mixed vigorously. The admixture was then wrapped in plastic bags and continuously mixed by shaking. Finally, the bags were stored until conducting the test. Considering ASTM D698^47^, the standard proctor compaction test was carried out to determine the OMC and Maximum Dry Density (MDD) of the VA-soil mixture (different VA contents). In these tests, no curing time was considered, and they were immediately carried out after preparation. Cylindrical specimens with 50 mm of diameter and a height of 100 mm were utilized for determining the Uniaxial Compression Strength (UCS) following the ASTM D2166^48^. It is worth noting that remolded samples were prepared with OMC based on the moist-tamped method^49^. All prepared specimens were again wrapped into plastic bags and maintained for several days inside a curing chamber with a controlled temperature of 23 ± 2 °C. In terms of saturated curing conditions, the samples were drowned into a pot full of water. A displacement control loading was applied with a rate of 1 mm/min for the uniaxial tests. This loading rate value corresponds to the deformation rate which is created beneath the pavement subgrades due to the traffic loads^38^.

**Figure 4:**
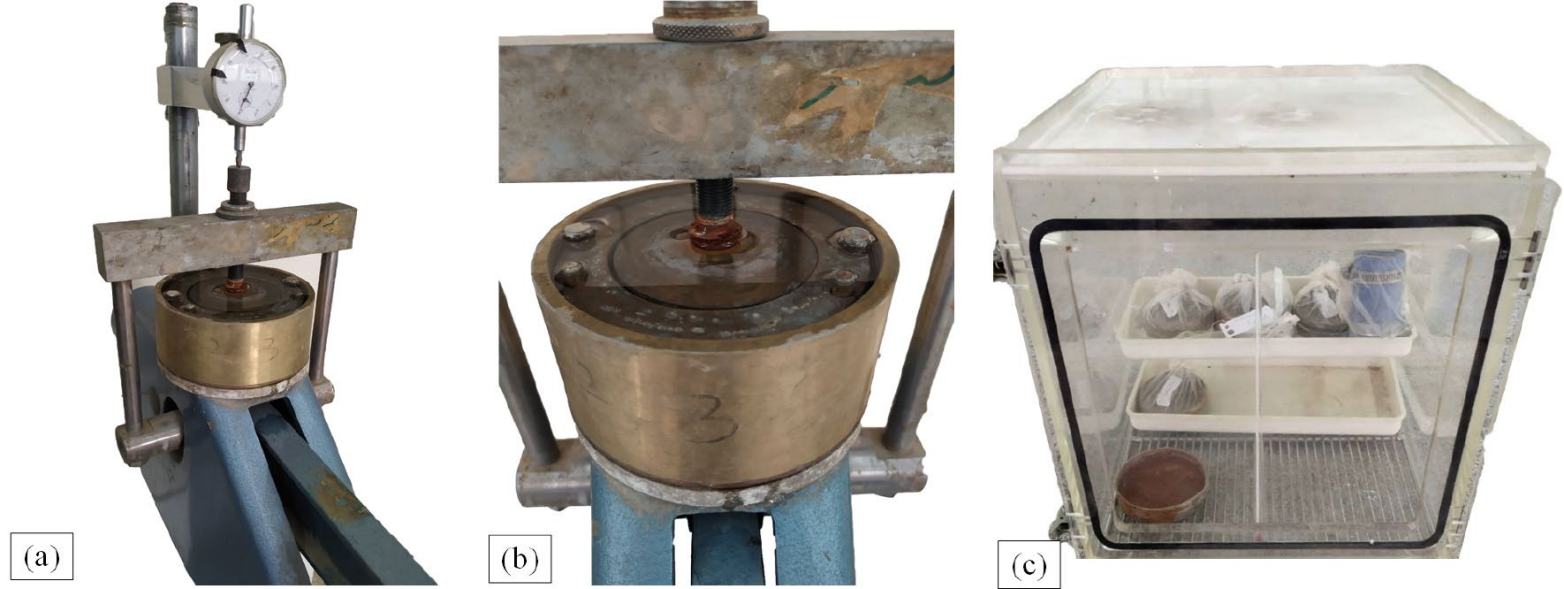
One-dimensional compression test; (**a**) under the loading application, (**b**) curing samples at saturated conditions, (**c**) curing sample at optimum moisture content (OMC) conditions.

One-dimensional compression tests were carried out on the stabilized soils regarding ASTM D2435^50^. A cylindrical mold of 75 mm in diameter and 20 mm in height was used for these tests. The specimen preparation was similar to the uniaxial compression tests, but the samples were placed in a curing chamber for 7, 14, 28, and 90 days. For each percentage of VA, two samples were reconstituted, i.e. for saturated and OMC testing conditions. Moreover, in terms of saturated condition, after assembling the porous stones on the top and bottom of the sample, the pot of the one-dimensional compression apparatus was brimmed with water and the temperature of the testing room kept constant at 23 ± 2 °C (see Fig. 4). The stress level in this tests is limited to 400 kPa which corresponds to secondary roads^37^.

## Results and discussions

### Effect of VA on OMC and MDD

**Figure 5:**
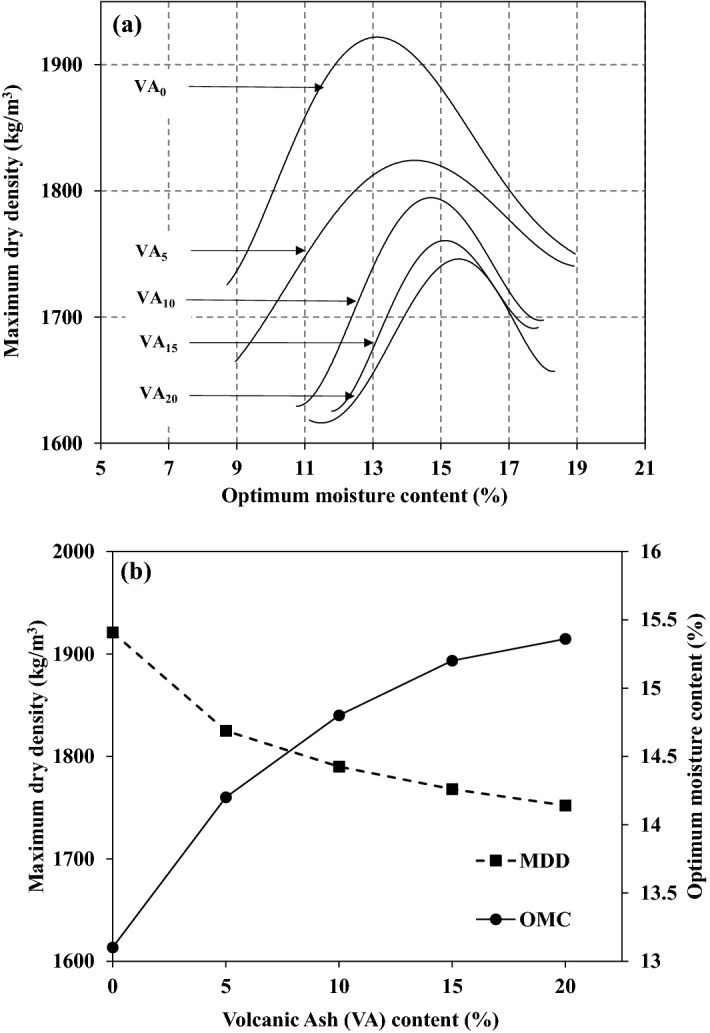
(**a**) Variation of the dry density with the moisture content, (**b**) variation of the maximum dry density (MDD) and optimum moisture content (OMC) with the volcanic ash (VA) content.

Figure 5a illustrates the dry density variation with the moisture content for different VA percentages. Figure 5b shows the results of the conducted standard proctor tests. The VA percentage impact on OMC and MDD is presented. By increasing the VA-percentage, from 0 to 20%, the MDD decreases approximately 9% (from 1920 to 1750 kg/m^3^). This trend can be attributed to the VA specific gravity and the grain size distribution of the mixture which was also observed by Hossain and Mol^38^.

**Figure 6:**
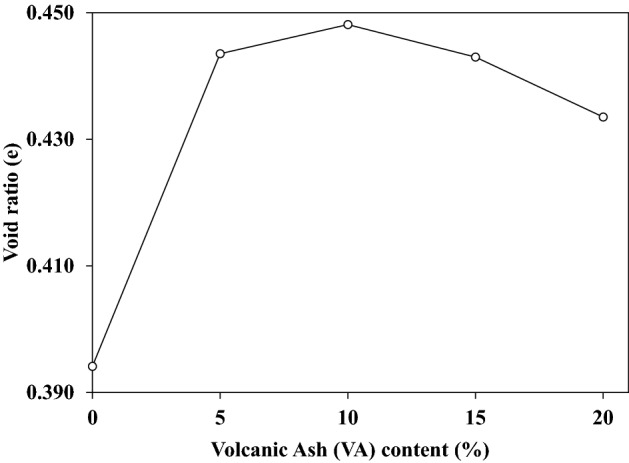
Variation of the void ratio with the volcanic ash (VA) content.

At first, additional VA powder coats the soil particles. It leads to coarser particles admixture and the free space volume increases consequently. However, the free space (void ratio) enlarges until the VA coats all particles surface. Then, more VA addition to the soil will fill free spaces, and the void ratio decreases^38^. The impact of adding more VA to the soil will then become low. This behavior can also be delineated by Fig. 6 which reveals the void ratio variation with the VA percentage. By increasing VA percentage, the void ratio increases of 10% and tends to decrease afterward. This reduction can be due to the VAs particle size.

An adverse trend is observed for the optimum moisture content variation versus the VA percentage. The OMC increases up to 2% by adding 20% of VA and the amount of water is in direct relationship with the VA content. As addressed by different researchers like Hossain and Mol^38^. They mentioned that this trend can be attributed to the VA-soil admixture water absorption for pozzolanic reactions.

**Figure 7:**
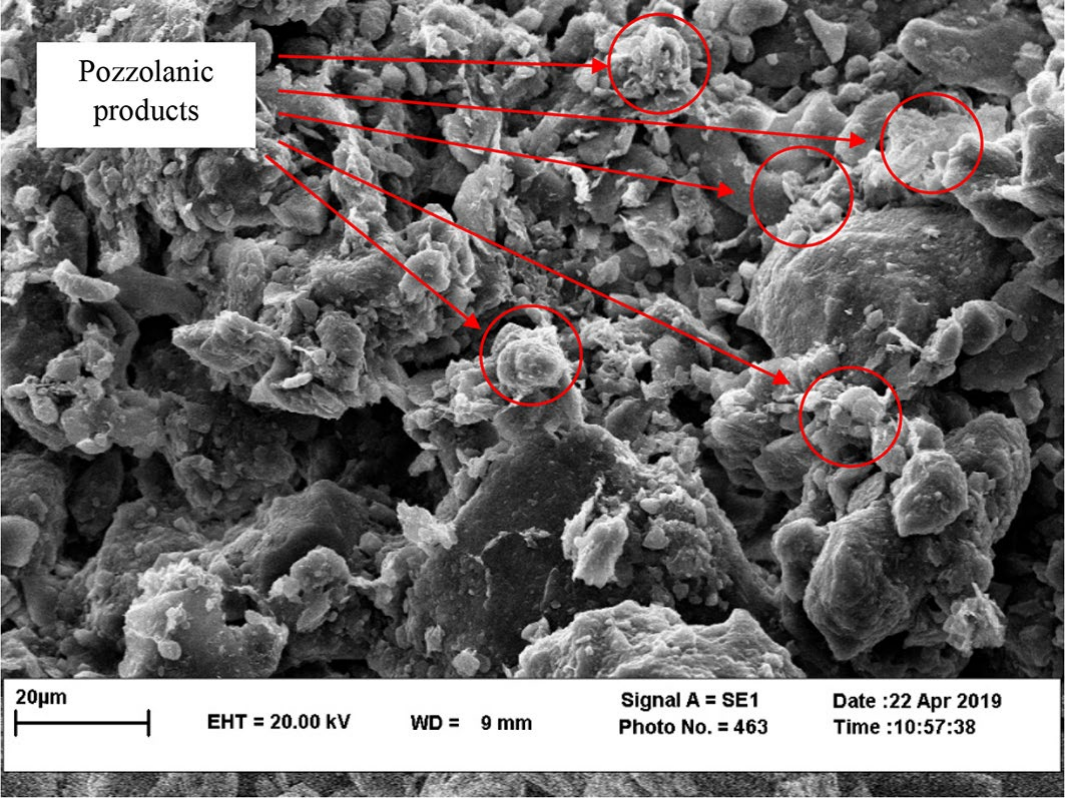
SEM micrograph of the VA_10_D_90_-S treated soil.

**Figure 8:**
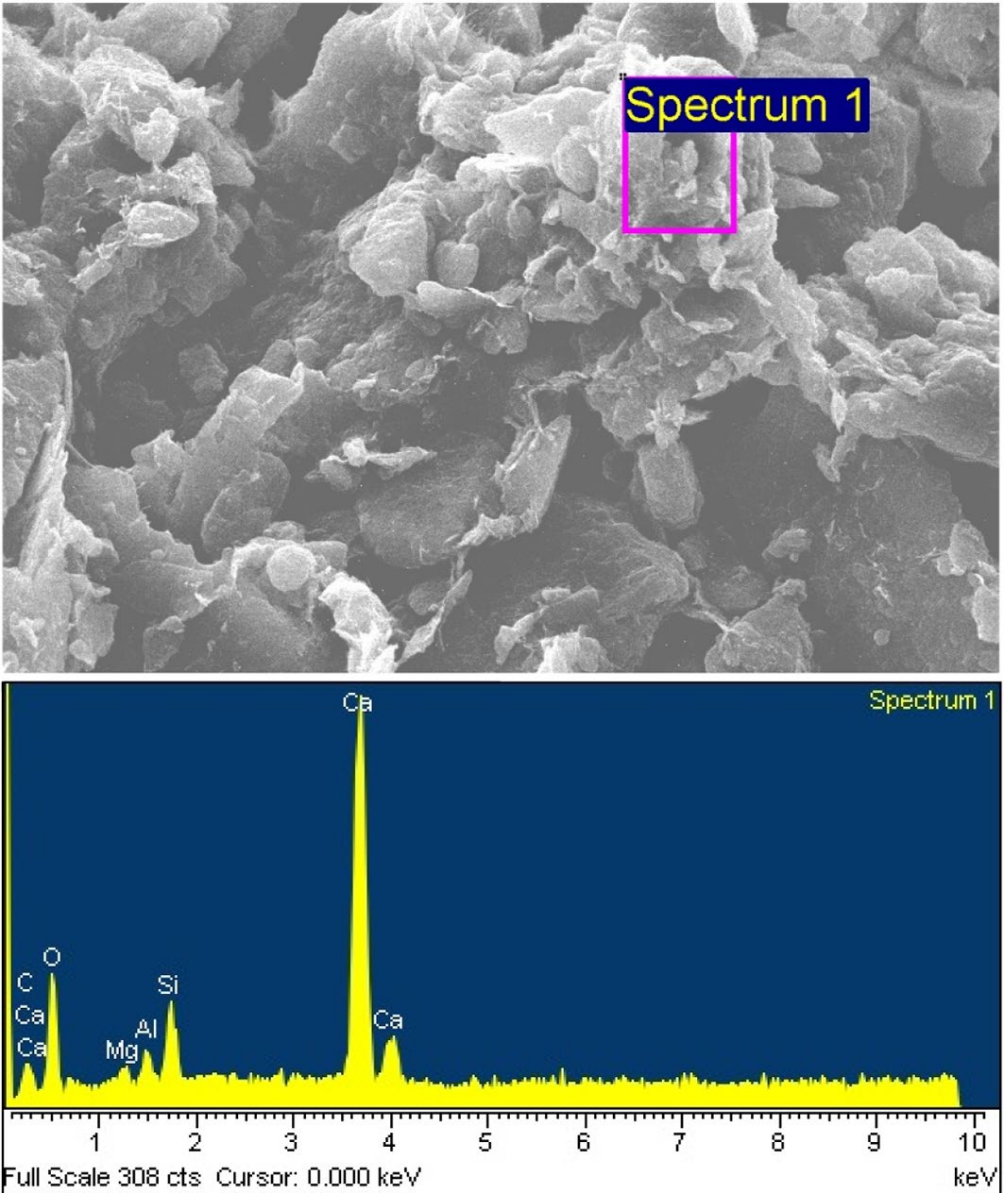
EDS graph of the VA_10_D_90_-S pozzolanic products.

Figure 7 illustrates the SEM photos for the VA_10_D_90_-S sample. Fine grains of VA cover the clay particles, and chemical bonds are fabricated. The chemical bonds contain CSH and CAH (bright spots in Fig. 7). An Energy Dispersive Spectroscopy (EDS) test was carried out to determine the compounds within the crystal bonds. As shown in Fig. 8, the amount of calcium found in CSH and CAH is high. Thus, the bright spots which are representing the solid bonds, are produced by the pozzolanic reaction^27^.

### Effect of VA on UCS

**Figure 9:**
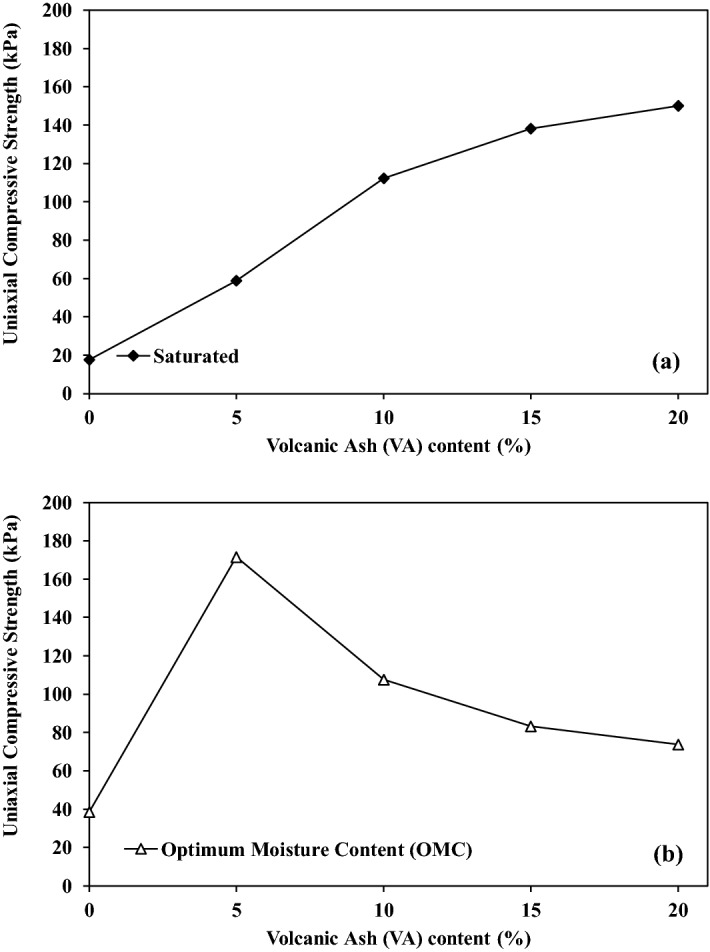
Influence of volcanic ash (VA) content on uniaxial compression strength (UCS); (**a**) at saturated condition, (**b**) at optimum moisture content (OMC) condition.

Figure 9 demonstrates the effect of adding VA on the UCS after 28 curing days. The UCS is around 17 kPa, while it raises up to 147 kPa for stabilized soils with 20% of VA. It represents an improvement of 760% once it is cured considering a saturated state (Fig. 9a). The presence of VA in the soil induces resistance bonds which leads to the soil cohesion enhancement and higher UCS.

On the other hand, UCS considering the OMC curing conditions increases as well, however, the largest strength appeared for VA_5_. Indeed, by adding 5% of VA to the soil, UCS starts from 45 kPa at VA_0_ and sharply increases to 170 kPa (277% of improvement) for VA_5_. Then it decreases non-linearly when adding extra VA to the soil, to approximately 74 kPa (64% of improvement) for VA_20_ (Fig. 9b). This trend was also been observed by^27^. The pozzolanic reactions cannot be completed due to the lack of water and this can make the situation worse by adding extra VA which leads to a higher void ratio in the soil sample. Moreover, VA particles are naturally non-cohesive, thus, by adding extra VA, the UCS decreases due to the absence of bonds.

### Oedometer modulus

**Figure 10:**
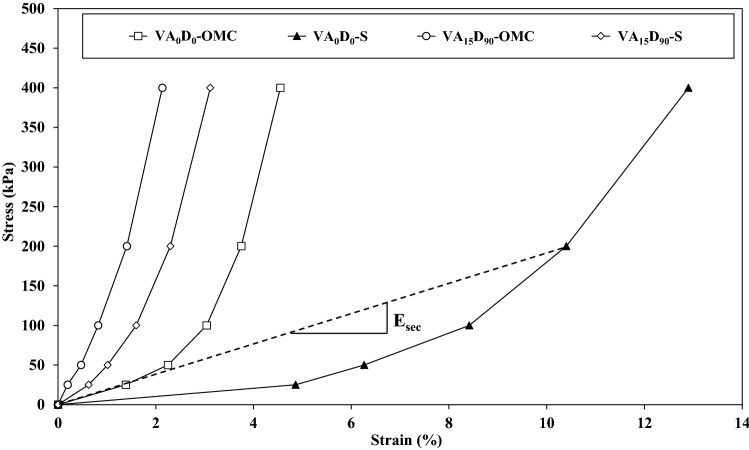
One-dimensional compression stress–strain curve.

A total of 36 1-D compression tests were conducted to investigate the VA addition effect on the oedometer modulus. To this aim, the secant elasticity modulus was determined for five stress levels, 25 kPa, 50 kPa, 100 kPa, 200 kPa, and 400 kPa. Figure 10 shows the stress–strain curves for four different tests, VA_0_D_0_-OMC, VA_0_D_0_-S, VA_15_D_90_-OMC, and VA_15_D_90_-S. For a constant stress level, by adding VA to the soil, the displacements become lower for both OMC and saturated conditions when compared with the non-stabilized soil case. The secant elasticity modulus is calculated according to the following equation (Eq. 1) and Fig. 10.

1 $${\text{E}}_{{{\text{sec}}}} = {\text{s}}_{{\text{v}}} /\varepsilon$$

In this study, a parameter is named IF, Improvement Factor. It can be obtained using the following equation:

2 $${\text{IF }} = {\text{ E}}_{{{\text{ss}}}} /{\text{E}}_{{{\text{ns}}}}$$

where E_ss_ is the oedometer modulus of the stabilized soil with VA and E_ns_ is the oedometer modulus of the non-stabilized soil (VA = 0%) for a constant stress level. IF indicates the settlement characteristics of the VA-soil mixture. In the following part, the effect of VA percentage, curing time, and condition (saturated or unsaturated) on IF are discussed.

#### Effect of the VA percentage on IF

**Figure 11:**
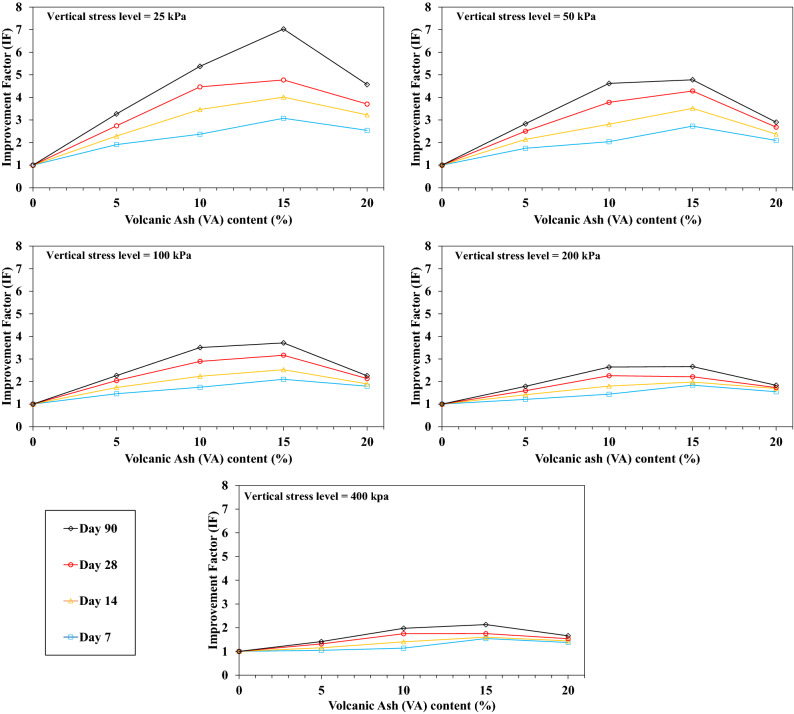
Influence of the volcanic ash (VA) content on the improvement factor (IF) at the optimum moisture content (OMC) condition.

Figure 11 shows the VA percentages variation versus IF for the OMC condition. Increasing the VA percentage until 15%, the IF increases for all the stress levels and declines for VA_20_. Indeed, for a higher amount of VA, the higher settlement is induced by VA_20,_ and IF reduces consequently.

**Figure 12:**
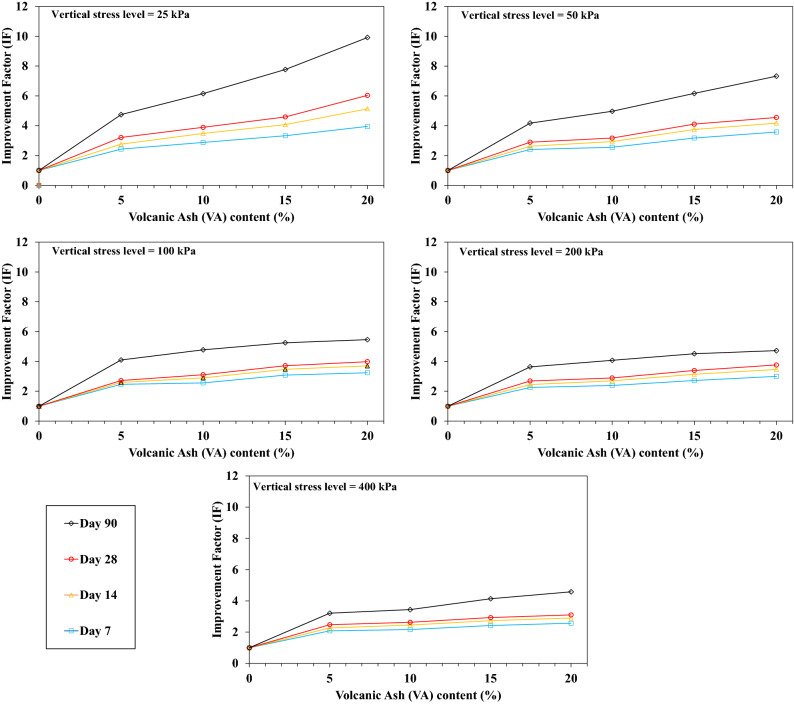
Influence of the volcanic ash (VA) content on the improvement factor (IF) for saturated conditions.

Figure 12 shows the results of the IF for the saturated one-dimensional compression tests (conventional consolidation tests). The more VA added to the soil, the more IF increases. Indeed, settlement decreases consistently with increasing the VA percentage for the saturated conditions. By comparing the results for both OMC and saturated conditions, the IF evolution can be attributed to the lack of water in the VA_20_. Hence, fabricated resistant bonds are smaller as in VA_15_. For the saturated condition, since the sample is brimmed with water, the VA pozzolanic reaction continues, and a higher IF is reached. The VA compressibility properties are higher than the soil one^30^; therefore, for unsaturated conditions, adding more VA from 15 to 20% requires more water for the pozzolanic reaction. Settlements are reduced for VA_20_ due to the existence of more VA in the sample.

#### Effect of the curing time on IF

**Figure 13:**
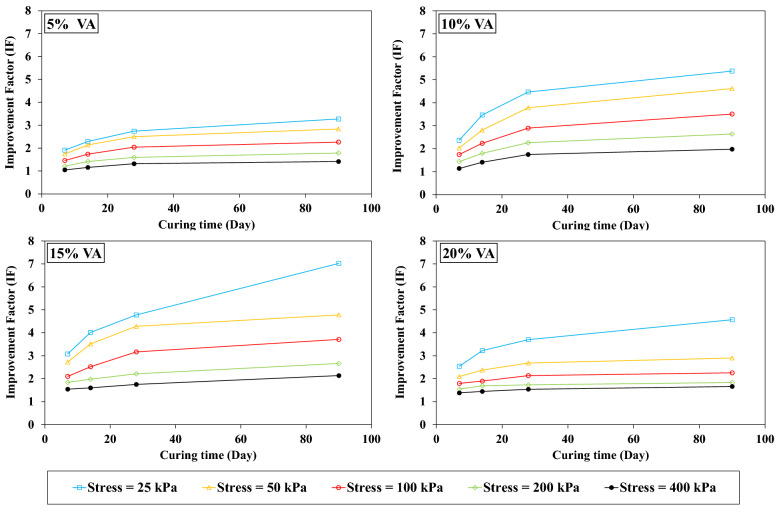
Influence of the curing time on the improvement factor (IF) for the optimum moisture content (OMC) conditions.

**Figure 14:**
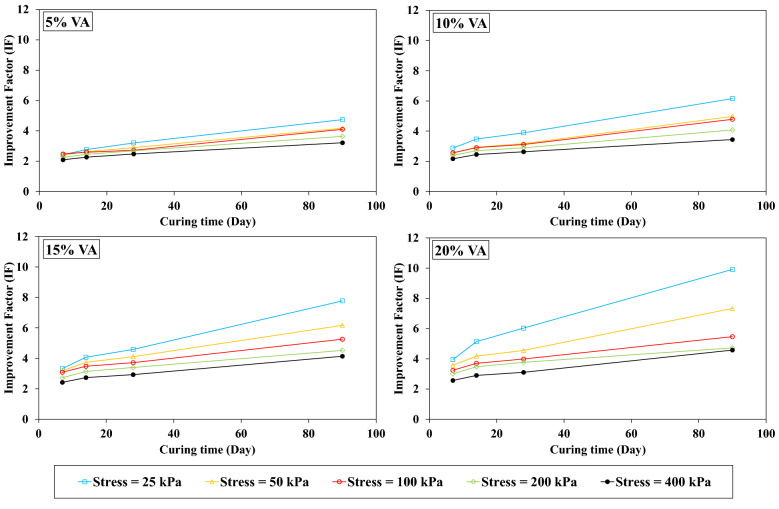
Influence of the curing time on the improvement factor (IF) for the saturated conditions.

As already stated, VA is a type of cementitious material, so the curing time (pozzolanic reaction period) has a remarkable impact on the samples strength. In the current study, the oedometer modulus of the VA-soil admixture are determined at 7, 14, 28, and 90 days of curing. Curings were conducted for OMC and saturated conditions, results are shown in Figs. 13 and 14, respectively. For both conditions, IF significantly increased. For instance, for a given stress level namely 25 kPa for VA_5_D_7_-OMC and VA_5_D_90_-OMC, IF raised up to 196% for the long-term curing period in contrast with its short-term curing time (IF_7 days_ = 2.6 and IF_90 days_ = 5.1). For the OMC states, due to the lack of enough water for the pozzolanic reaction, the IF remains almost constant after 30 days of curing time. While, IF increases relatively linearly by increasing the curing time for the saturated state. The pozzolanic reaction requires water, and the more water there is, the more resistance bonds are produced, so that at saturated conditions, IF considerably increases until 90 days of curing time.

#### Stress level effect on IF

**Figure 15:**
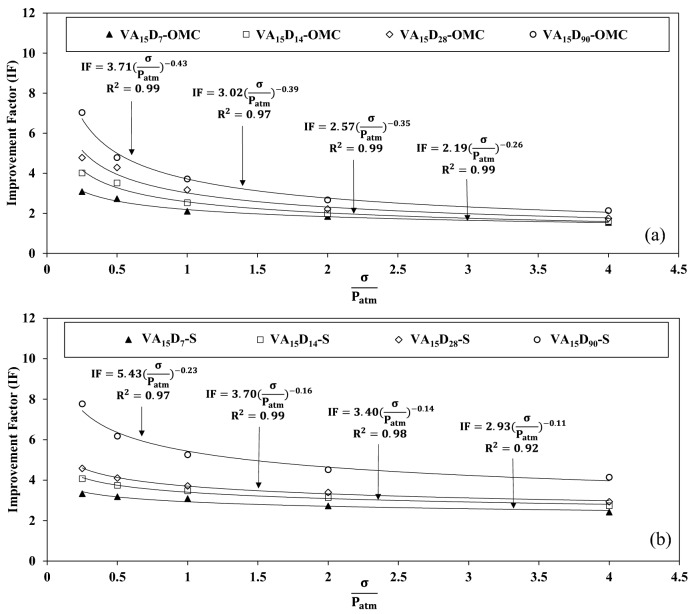
Improvement factor (IF) changes with the stress level for 15% additional of volcanic ash (VA_15_) for different states; (**a**) optimum moisture content (OMC), (**b**) saturated.

As stated previously, the oedometer modulus is determined for five different stress levels. Figure 15 a,b indicate the IF changes with various stress levels for VA_15_ in OMC and saturated conditions. An indirect nonlinear relationship between the stress level and IF is observed for all the curing times. The IF decreases dramatically with the stress level increase. The bonds between the soil and VA which were fabricated during curing time can be fractured under the loading application. Indeed, the higher the load level, the more bonds are broken. It can also be seen in Fig. 15 that the relationship between the IF and stress level can be interpolated as a power function, $IF=k{(\frac{\sigma }{{P}_{atm}})}^{n}$, and the measures of the R-squared value indicate a good fit for the data. In the interpolated function, *k* and *n* are the constants which vary due to the curing condition, time, and VA content. These parameters, *k* and *n*, respectively vary based on the curing condition and stress level.

**Figure 16:**
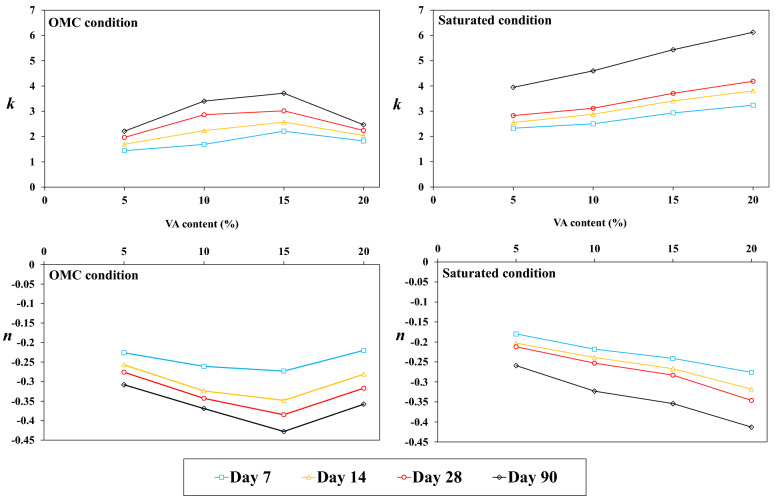
Influence of the volcanic ash (VA) content and curing time on *n* and *k.*

As indicated in Fig. 16, the variation of *k* and *n* with the percentage of VA shows an analogous trend with Figs. 11 and 12. Additional VA expands the fabricated bonds which make the VA-soil mixture more brittle and leads to the higher values of *k* except for VA_20_-OMC. Therefore, this fragility causes more cracks appearance as the consequence of the stress application. Also, *k* values are greater for the saturated conditions rather than OMC states due to the presence of more pozzolanic bonds. In fact, the key parameters that affects *k*, are the VA percentage and curing conditions (time and moisture) and it depends on the formation of bonds. *n* has negative values which indicates the reduction effect of additional VA for higher stress levels. Likewise, *n* values are lower for saturated conditions. Because the more the moisture content is, the less the clay behavior is brittle. Therefore, samples prepared using OMC conditions are more brittle than the saturated state, and *n* has a greater value for the OMC conditions.

### Effect of VA on the consolidation parameter

**Figure 17:**
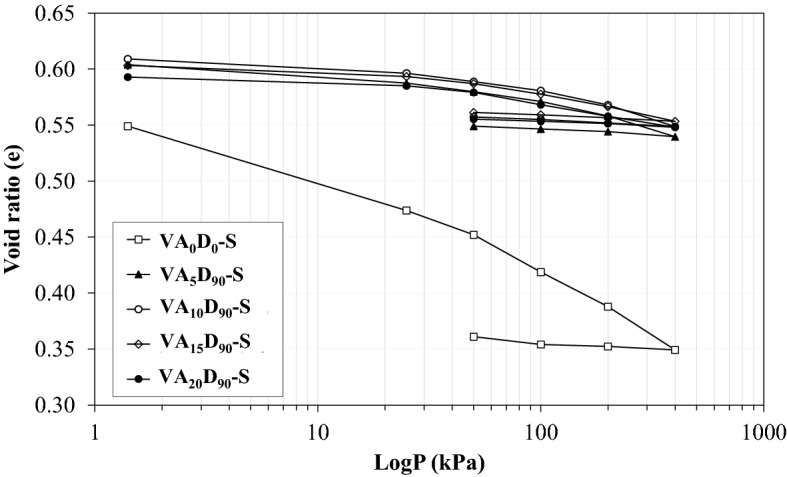
Consolidation curve for different volcanic ash (VA) contents at 90 days of curing time.

**Figure 18:**
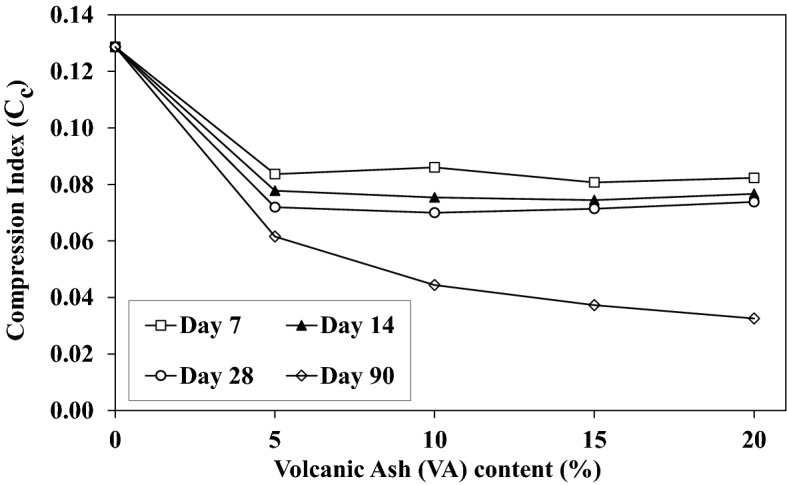
Influence of the volcanic ash (VA) on the compression index (C_c_) with the curing time.

Figure 17 shows the one-dimensional compression tests at saturated conditions (conventional consolidation tests). Figure 17 presents the typical consolidation test graph for different percentages of VA at 90 days of curing time. As stated previously, additional VA increases the void ratio which leads to shifting the graph up. The Compression Index (C_c_) shows the capability of the soil to decrease its volume under external loads. Figure 18 illustrates the variation of C_c_ with the VA percentage at different curing times. As seen, adding VA to the soil decreases the C_c_, while more VA influences C_c_ for the short-term curing time. This effect becomes important when the sample is cured for 90 days. Shaped bonds are fragmented by additional VA for short-term curing time. This short-term curing period trend can be attributed to the fact that resistance bonds are broken at high stress levels and C_c_ is determined at the final increment of loading (tangential slope at 400 kPa stress). In terms of 90 days curing time, for example, C_c_ decreases down to 60%, from 0.082 to 0.032, for VA_20_-D_7_-S and VA_20_-D_90_-S.

**Figure 19:**
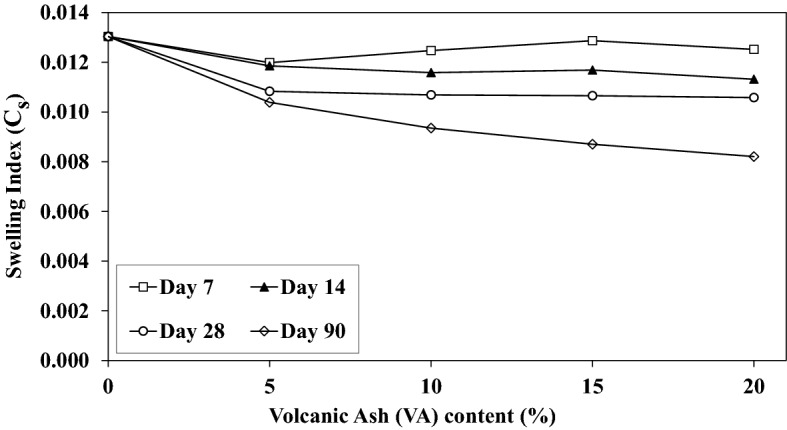
Influence of the volcanic ash (VA) content on the swelling index (C_s_) for various curing time.

**Figure 20:**
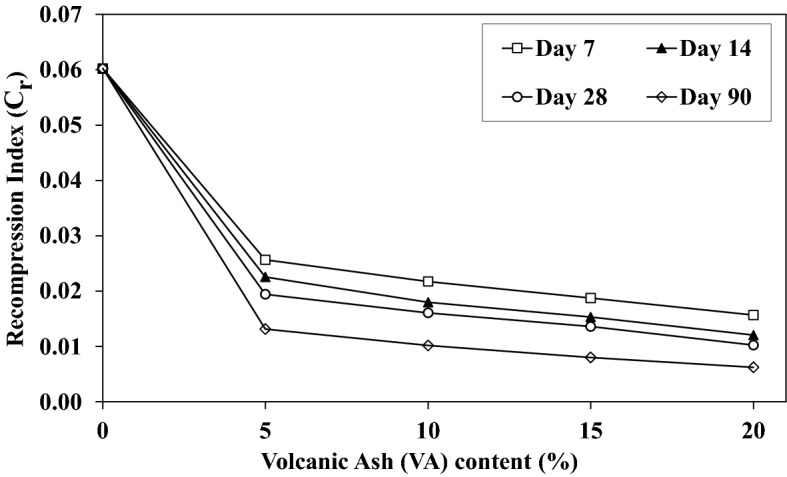
Influence of the volcanic ash (VA) content on the recompression index (C_r_) for different curing times.

The swelling coefficient (C_s_) which reveals the capability of the soil to increase its volume after unloading. Figure 19 shows changes of C_s_ with the VA content. Correspondingly, VA has a negligible impact on C_s_ and exclusively for short-term curing time. Nevertheless, C_s_ decreases for long-term curing times with increasing the VA percentage. Pozzolanic bonds hinder swelling to a limited extent when they are well fabricated. More specifically, the Recompression Index (C_c_ determination at the initial increment of loading, C_r_), is also assessed to elaborate on the effect of additional VA on settlement properties. Figure 20 indicates the C_r_ changes with VA content. C_r_ decreases with the VA increase. This reduction becomes linear by adding extra VA. Therefore, it can be stated that addition of VA to the soil is effective for consolidation parameters at lower stress levels.

## Conclusion

In this study, the feasibility of using Volcanic Ashes (VA) for clayey soils stabilization is investigated by employing compaction, uniaxial compression, and one-dimensional compression tests. Four different percentages of VA (i.e. 5, 10, 15, and 20%), were added to the soil in order to study the VA effect on the uniaxial strength as well as on the oedometer modulus at the Optimum Moisture Content (OMC) and fully saturated conditions during curing periods varying from 7 to 90 days. The impact of VA on consolidation parameters was evaluated. The laboratory investigations allow to obtain the following conclusions:

- Using VA increases the void ratio and OMC. This can be attributed to the space and bond formation after the pozzolanic reaction. Therefore, additional VA increases the water absorption,
- For the OMC conditions, the optimum percentage of additional VA is respectively equal to 5% and 15% for the uniaxial and 1-D compression tests. While, for saturated curing conditions, no optimum VA was observed for both tests. In other terms, it was found that the presence of water allows the production of the bonds formation,
- The soil stiffness parameters are significantly improved by adding VA, and this improvement is more important for saturated curing conditions,
- Concerning the curing periods, for a constant percentage of VA, short-term curing condition (7 days) increases the Improvement Factor (IF). Nevertheless, the enhancement is more remarkable for long-term curing conditions (14 days and more). Indeed, more bonds are fabricated by the pozzolanic reaction throughout the curing time,
- In terms of stress level, it can be stated that using VA would be more productive for low service loads. Otherwise, the fabricated bonds fragment, hence, both stiffness and resilience are diminished. Therefore, stabilization with VA would be functional for pavement design in contrast with foundation-based improvement,
- The addition of VA decreases the Recompression Index (C_r_) more dramatically than the Compression Index (C_c_). It has a low impact on the Swelling Index (C_s_). Adding extra additional VA has a negligible effect on the consolidation parameters at short-term (up to 28 days) and it becomes considerable for long-term (i.e. 90 days).

## Data Availability

All data generated or analyzed during this study are included in this published article. The raw data is also available from the corresponding author on reasonable request.

## Abbreviations

VA: Volcanic ash
CSH: Calcium silicate hydrate
CAH: Calcium aluminate hydrate
SEM: Scanning electron microscopy
EDS: Energy dispersive spectroscopy
OMC: Optimum moisture content
MDD: Maximum dry density
IF: Improvement factor
UCS: Uniaxial compression strength

## List of symbols

VA_n_D_t_-OMC: N percentage of volcanic ash–soil mixture cured in t days at optimum moisture content condition
VA_n_D_t_-S: N percentage of volcanic ash–soil mixture cured in t days at the saturated condition
E_sec_: Secant modulus of elasticity
_σ__v_: Vertical stress
ε: Vertical strain
E_ss_: Oedometer modulus of stabilized soil
E_ns_: Oedometer modulus of non-stabilized soil
k: Constant parameter
n: Constant parameter
C_c_: Compression index
C_s_: Swelling coefficient
C_r_: Recompression index
